# Tailored Basic Life Support Training for Specific Layperson Populations—A Scoping Review

**DOI:** 10.3390/jcm13144032

**Published:** 2024-07-10

**Authors:** Sebastian Schnaubelt, Christoph Veigl, Erwin Snijders, Cristian Abelairas Gómez, Marco Neymayer, Natalie Anderson, Sabine Nabecker, Robert Greif

**Affiliations:** 1 Department of Emergency Medicine, Medical University of Vienna, 1090 Vienna, Austria; 2 PULS—Austrian Cardiac Arrest Awareness Association, 1090 Vienna, Austria; 3 Department of Emergency Medicine, Antwerp University Hospital, 2650 Edegem, Belgium; 4 Emergency Medical Service Vienna, 1030 Vienna, Austria; 5 Faculty of Education Sciences and CLINURSID Research Group, Universidade de Santiago de Compostela, 15705 Santiago de Compostela, Spain; 6 Simulation and Intensive Care Unit of Santiago (SICRUS) Research Group, Health Research Institute of Santiago, University Hospital of Santiago de Compostela—CHUS, 15706 Santiago de Compostela, Spain; 7 Faculty of Medical and Health Sciences, University of Auckland, Auckland 1023, New Zealand; 8 Department of Anesthesiology and Pain Management, Mount Sinai Hospital, Toronto, ON M5G 1X5, Canada; 9 Faculty of Medicine, University of Bern, 3012 Bern, Switzerland; 10 School of Medicine, Sigmund Freud University Vienna, 1020 Vienna, Austria

**Keywords:** specific populations, basic life support, BLS, cardiopulmonary resuscitation, CPR, tailored, adapted, education, training, teaching, out-of-hospital cardiac arrest, OHCA

## Abstract

**Background:** Basic life support (BLS) is a life-saving link in the out-of-hospital cardiac arrest chain of survival. Most members of the public are capable of providing BLS but are more likely to do so confidently and effectively if they undertake BLS training. Lay members of the public comprise diverse and specific populations and may benefit from tailored BLS training. Data on this topic are scarce, and it is completely unknown if there are any benefits arising from tailored courses or for whom course adaptations should be developed. **Methods:** The primary objective of this scoping review was to identify and describe differences in patient, clinical, and educational outcomes when comparing tailored versus standard BLS courses for specific layperson populations. This review was undertaken as part of the continuous evidence evaluation process of the International Liaison Committee on Resuscitation. **Results:** A primary search identified 1307 studies and after title, abstract, and full-text screening, we included eight publications reporting on tailored courses for specific populations. There were no studies reporting direct comparisons between tailored and standardized training. Seven (88%) studies investigated courses tailored for individuals with a disability, and only one study covered another specific population group (refugees). Overall, the quality of evidence was low as the studies did not compare tailored vs. non-tailored approaches or consisted of observational or pre–post-designed investigations. **Conclusions:** Tailored BLS education for specific populations is likely feasible and can include such groups into the pool of potential bystander resuscitation providers. Research into comparing tailored vs. standard courses, their cost-to-benefit ratio, how to best adapt courses, and how to involve members of the respective communities should be conducted. Additionally, tailored courses for first responders with and without a duty to respond could be explored.

## 1. Introduction

Community first responders can provide a vital link in the chain of survival for out-of-hospital cardiac arrest (OHCA) by providing basic life support (BLS), bridging the time delay to advanced life support (ALS) and definitive treatment [1]. Confident and competent BLS is more likely when first responders have received previous training [2]. Numerous initiatives around the world have been developed to increase the proportion of lay populations who are trained in BLS. This includes standardized BLS courses offered by the European Resuscitation Council (ERC) [3] and the American Heart Association (AHA) [4], as well as awareness campaigns like World Restart a Heart [5] and Kids Save Lives [6]. However, when providing education to diverse populations, it is possible that a standardized approach is suboptimal for some learners, and tailored training may serve the needs of specific groups better, for example, for those with differing physical abilities or professional backgrounds. Little is known about on how to tailor BLS courses, and this is the first review to locate and describe interventional research in this area. The purpose of this review is to provide an overview of what is known about tailored basic life support education for specific lay groups, including the targeted groups, the nature of tailoring, and any positive or negative effects on learning and skills, while also revealing gaps in existing knowledge and opportunities for further research. 

## 2. Materials and Methods

### 2.1. Protocol

This review was undertaken as part of the International Liaison Committee on Resuscitation (ILCOR) continuous evidence evaluation process with engagement of five members from the Education, Intervention and Teams (EIT) Task Force (SS, CAG, NA, SN, RG) and three external content experts (CV, ES, MN). A specific review protocol including a search strategy was agreed upon by the EIT Task Force, reflecting the current ILCOR processes for scoping reviews [7]. This review follows a recommended methodological framework [8] and the Preferred Reporting Items for Systematic reviews and Meta-Analyses (PRISMA) checklist for scoping reviews [9]. The PRISMA checklist can be found in Supplement S1. 

### 2.2. The PICOST Question

We followed the format of Population, Intervention, Comparator, Outcome, Study Design and Timeframe (PICOST), which was defined as follows (definitions are provided below):

- Population: specific adult layperson populations and/or groups participating in BLS training.
- Intervention: tailored BLS training.
- Comparison: non-tailored BLS training.
- Outcomes: patient outcomes (critical): ROSC, survival to hospital discharge, 30-day survival, 12-month survival, neurological outcome. Clinical outcomes (critical): starting CPR in case of real cardiac arrest; performance during real CPR. Educational outcomes (important): knowledge and skill acquisition, willingness to perform CPR, barriers, and enablers towards performing CPR, participant satisfaction and/or knowledge as well as skill retention at the end of the respective course and later (e.g., 3 months, 1 year), implementation success, resource implications, and cost effectiveness.
- Study Design: randomized controlled trials (RCTs) and non-randomized studies (non-randomized controlled trials, controlled before-and-after studies, cohort studies, and case series n ≥ 5), reviews, and surveys in respective population groups with at least an abstract in English were eligible for inclusion. Research was aimed at teaching BLS to children; research on CPR training for different healthcare professionals were excluded, as both were sufficiently covered elsewhere.
- Time frame: from inception to 21st of February 2024.

The following definitions respective to the PICOST were agreed upon within the EIT Task Force:

- “Specific”: We defined “specific population and/or group” as a subgroup of the general population having a specific feature (e.g., a specific job, an age-group, etc.). We acknowledge that this is a very wide definition.
- “Layperson”: We defined “layperson” as the general adult population excluding qualified, retired, or in-training healthcare professionals (e.g., medical students, nursing students, paramedic students, etc.). However, to make the approach more structured, we defined two groups of laypersons:
  ○ Duty to respond: Laypersons (non-healthcare professionals) that *do* have a duty to respond. This includes any type of professional first responders (e.g., law enforcement, firefighters), lifeguards, flight crews, and any other people that would have a duty to attend to victims in an emergency.
  ○ No duty to respond: Community laypersons that have *no* duty (occupational expectation) to respond to a cardiac arrest. This includes anyone else not included in the group mentioned before and trained community first responders who would respond to an alarm on a smartphone app or similar (as they do not have an occupational duty to respond).
- “Standard BLS training” or “non-tailored BLS courses” are considered BLS courses that follow current recommendations from large course developers and organizers (e.g., AHA, ERC) without changes intended to meet the needs of specific learner populations.
- “Tailored training” or “tailored courses”: altered to serve the specific needs of a population (e.g., in duration, frequency, content, assessment, feedback, used material and devices, specific aids, contextualization of the environment, specially trained instructors, etc.).

The original PICOST question was asked for studies reporting on tailored courses for specific populations and a comparison was made between tailored courses and standard courses. However, the found publications only reported on adapted courses comparing these adaptations to standard courses or other adaptations in a specific population. None of the studies reported on courses specifically tailored to that specific population. After a Task Force discussion, we decided to broaden our inclusion criteria to any reports on courses specifically tailored to specific populations, even if there was no comparison to serve as evidence for this scoping review. We thus applied the following criteria: 

Inclusion: publications reporting on BLS courses that were adapted/tailored specifically for a population group. 

Exclusion:

- Studies only assessing CPR knowledge and/or skills in a specific population without an adaptation of the course to meet the needs of that specific population.
- Comparisons of different instructional designs not being tailored to a specific population. Example: comparing video-based versus instructor-based CPR education in university students, without being tailored to university students.
- Research which describes BLS education tailoring but is not of an interventional or experimental design.
- Studies on participants less than 18 years.
- Studies involving high-risk patients and/or their relatives, as this topic is already covered by another ILCOR review [10].
- Studies reporting on chest-compression-only CPR as the sole adaptation in their courses, as this is often already regarded as standard in layperson training.

### 2.3. Search Strategy and Selection Process

The search strategy was performed by information specialist Mary-Doug Wright (AHA, Dallas, TX, USA) and peer-reviewed by a second one (Medical University of Vienna, Austria; asked not to be named)—see Supplement S2. Records from database searches were downloaded and imported into an EndNote database to facilitate the removal of duplicates. Databases searched included Embase, MEDLINE(R) ALL (multi-database search via Ovid), and Cochrane Central Register of Controlled Trials (Cochrane Library via Wiley Online). Final database searches were conducted in July 2023. An updated search on the 21 February 2024 resulted in no additional relevant publications. See Supplement S2 for the full search strategy. A search of grey literature was not performed. 

In total, 1203 abstracts were imported in Rayyan (https://www.rayyan.ai/) and screened independently by the authors of this scoping review. Moreover, 104 additional abstracts which were found due to cross-citations in the reviewing process being added by the reviewers, leading to a total of 1307 screened abstracts. Conflicting decisions were resolved via an agreement between the reviewers. A total of 17 duplicates were deleted, and 74 articles were selected for full-text retrieval. After assessing the full-text contents of the papers, 66 publications were excluded (due to them not reporting anything covered by the original or adapted PICOST), leaving 8 studies included in this review.

To identify the resource perspective of the publications, we applied the World Bank definition to classify the countries of origin into four categories by gross national income per capita, namely low-income economies, lower-middle-income economies, upper-middle-income economies and high-income economies [11].

## 3. Results

We included eight publications that originated from diverse geographical areas, with most of them being from Europe (Table 1). The majority (n = 7, 88%) came from high-income countries, and none came from low-income countries (Table 1). Except for one study [12], all studies were published within the recent ten years.

Table 2 summarizes the included studies and respective findings. Seven (88%) studies [12,13,14,15,16,17,18] investigated courses that were provided for individuals with a disability, and only one study [19] covered another specific population group (refugees). The specific learner groups for which education was tailored were those with Down syndrome [13,16], blindness [14,15], and deafness or hearing impairment [12,17,18]. 

No studies with tailored courses for specific populations compared their tailored approach to standard courses. However, as only a limited number of studies on tailored courses was found, we also included ones without this comparison, as mentioned above.

After completing BLS courses, especially ones tailored to individuals with Down syndrome, the respective participants were able to perform BLS, including AED use. These performances were not worse than the ones seen in other laypersons’ BLS courses. Tailoring for this special group of providers meant paying special attention to shorter sessions due to a potentially reduced attention span and introducing “lightweight” educational material such as videos with comic elements. Both studies used chest-compression-only CPR [13,16]. 

Two studies assessed CPR education for the blind: The first study focused on “training adapted to the participants’ needs” combined with chest-compression-only CPR, with results comparable to other BLS providers [14]. Two years later, the “tailoring” was refined and included supervisors with special pedagogic training and a very “tactile approach”. The CPR scenarios were performed successfully, except for low chest compression quality. This tailored BLS training also included rescue breaths [15].

There were three studies describing training tailored to learners with hearing impairments [12,17,18]. All three studies incorporated a sign language interpreter in their tailoring approaches, and all three did not alter the classic 30:2 approach (thus also teaching rescue breaths). Activating the EMS and following the voice prompts of an AED were seen as the most challenging learning points for first responders with a hearing impairment [12,17,18]. Strnad et al. also tailored a general BLS approach, incorporating slight adaptations like sending a text message to a respective emergency service for people with a hearing impairment [17].

One further study addressed BLS education for refugees: tailoring the courses consisted of having translators for the respective native languages on site—providing a special focus on general health literacy— and additionally teaching chest-compression-only CPR (it is, however, debatable whether chest-compression-only CPR in itself would be considered as tailoring) [19].

Despite a scoping review having no systematic risk of bias and certainty of evidence assessment, we generally found that the quality of evidence of the included studies tended to be low. The comparative studies did not compare tailored vs. non-tailored approaches, and the other studies were observational or pre–post-designed. Three of the included studies were reported as research letters [13,14,18], which provided only limited information.

## 4. Discussion

The EIT Task Force reviewed this topic in response to the awareness of BLS training adaptations—for instance, for individuals with a disability [20]—and the potential effects of tailoring on BLS learning and skills. With an expanding focus on systems to save lives, including community first responders, public-access AEDs, and increasing bystander CPR rates, ways to further enhance survival outcomes must be sought [21]. Disparities in layperson resuscitation education are known [22], and specific populations who are not healthcare providers may require specific BLS training due to their individual backgrounds (e.g., working in a special environment or having special needs or visual impairments) [1,23]. Specific groups within communities may be willing to attend tailored BLS courses and provide CPR but may not be served by standardized CPR courses. 

Interestingly, we found no studies comparing tailored courses to standard BLS courses, which was the intention of the original PICOST question. It thus remains unanswered if tailored BLS education for specific population groups compared to standard approaches can produce different results. However, summarizing the included data without such a comparison allowed us to provide a current overview of tailored courses for specific populations.

The studies reported only limited details about how the courses were tailored for the needs of the specific groups. Rather, somewhat adapted courses were conducted to show the feasibility of CPR education in the respective groups. Also, none of the studies provided a detailed insight into the development of their tailored course and even less so into the potential participation of members of the addressed groups in the specific content development.

We acknowledge that educators will often make (and probably have always made) small adaptations in courses to meet the individual needs of participants without conducting an educational study around it. This will most likely not be called tailoring and is rarely reported in scientific publications. However, “real” tailoring needs to systematically address the needs of learners, the potential teaching barriers, and the enablers towards optimal performance. All that should be embedded in a structured approach and validated to ensure the most beneficial effect for learners. To judge that, comparative studies on standard BLS courses are needed. 

The definition of a “standard”, non-tailored, BLS course is not easy, especially from the perspectives of lower resource settings which pose numerous challenges to the “standard” ways of teaching BLS [24]. For this review, we used a “standard” instructor-led manikin-based course based on the current guidelines from the AHA or ERC. However, modern blended learning formats [25] have the potential to develop specific tailored courses within the frame of the current teaching approaches from regional resuscitation councils. 

We also acknowledge that the benefits displayed by tailored courses could stem from specific population groups being educated together with their peers. This is a potential source of bias that needs to be kept in mind for future research. 

Despite the studies found for this review, several other specific populations could potentially benefit from tailored training (not an exhaustive list), as follows:

- Low socioeconomic background: Certain resource settings might lack minimum BLS standards, and location-specific solutions could be developed together with local experts [26,27]. A one-size-fits-all approach may not be sufficient to promote “CPR readiness” in deprived communities, and future approaches to working with disadvantaged communities could be tailored to local communities [28,29,30,31]. For instance, the location of publicly available training plays an important role [32], and targeted CPR training for low-education and low-income neighborhoods may increase bystanders’ CPR capabilities and improve OHCA outcomes [33,34]. As there is often a lack of any CPR-related courses in certain areas, shortened or cheaper courses could potentially provide an opportunity to attract more participants [35].
- Police or firefighters: Time to defibrillation decreased and survival from out-of-hospital cardiac arrests increased with the implementation of police and firefighter BLS programs [36,37,38,39,40]. Chest-compression-only BLS training may be more suitable for police when they are the first responders [41], and the interval between a call being received by them and for them to arrive on scene should be reduced by focusing on improvements in communication [42]. However, it is entirely unclear whether a more tailored training approach (than just chest-compression-only CPR) might bring additional benefits.
- Schoolteachers: Schoolchildren are considered a target population for receiving BLS education, and schoolteachers have been pointed out as the best option to teach them about it. It thus seems reasonable to teach schoolteachers about CPR at universities during their initial education [6,43,44]. However, questions such as how long the training should be or who could perform the respective teaching to the teachers have not been sufficiently answered yet. A tailored training approach could be designed for schoolteachers since they have different characteristics than the general public; for instance, they have already learned didactics and training methodologies [43].
- First responders with no “duty to respond”: First responders are not always required to respond to cardiac arrests as part of their jobs. Rather, first responders could also comprise people who simply have a first aid certificate and are registered in a first responder app. The literature on this is very heterogenous (because it basically comprises all publications, including first responders, ever). Tailored courses could serve as in-between CPR education.
- Lifeguards and/or boat crews: Lifeguards may need specific course topics and more regular follow-up training [45,46]. Boat crews may or may not benefit from courses with a lower emphasis on AED use [47,48].
- Elderly People: specific first aid courses including BLS training may improve educational outcomes in elderly individuals, willingness to perform CPR, and patient outcomes [30,49,50,51].
- Gender: the impact of gender on BLS attitudes and performance shows contradicting results in the literature, and it is unclear whether specific approaches and/or specially tailored programs should be considered [27,50].
- Individuals with various kinds of impairments / disabilities: Individuals with disabilities cannot just be excluded from various activities of social life, including CPR training. Various subgroups might benefit from tailored training [20].
- Migrants or Refugees: Population groups in society comprised of migrants and/or refugees coming from different cultural backgrounds and speaking various foreign languages comprise a considerable fraction of today’s general population in many countries. BLS courses for these groups could need tailoring [19,52].
- Specific sports groups: For instance, surfers [53] or football players could benefit from tailored BLS training. Sports groups are also potentially highly influential as ambassadors for advertising the message of saving lives across to a large population [54,55].
- Volunteers at long-distance races (e.g., running, cycling, triathlon, etc.): Although there is a low overall risk of cardiac arrest during running races, the number of participants in marathon and half-marathon races is increasing annually, and there are numerous reports of race-related cardiac arrest. However, there are often thousands of spectators and volunteers that could help during emergencies at such events, offering the opportunity of employing mass training with special tailored BLS courses [56].
- Flight crews: Flight crews are regularly exposed to a very heterogenous group of passengers. Guidelines on in-flight cardiac arrest have been developed; however, data on tailored training programs for them attending the cardiac arrest are scarce. Also, in the unlikely event of cardiac arrest in space, special circumstances presented by microgravity and spaceflight must be considered with relation to central points, such as the rescuer’s position, the methods used for performing chest compressions, airway management, and defibrillation. Moreover, in this area, the literature lacks suggestions for tailored training [57,58].
- Higher-education students: Tertiary students (>18 years old) who are not training to become health professionals are an important specific target group for BLS courses. However, whether their learning needs may be better met through tailored courses is unclear. Nonetheless, they form a quite large and important population group in almost every country worldwide, are young, and thus may be potentially eager to act in the case of an emergency. Also, they may be reached easily because they are associated with tertiary institutions [59,60].
- Other specific groups: prisoners may be open to CPR training [61].

This information provides an opportunity for a wide field of curriculum development and research to be carried out, as no sufficient evidence was found in the current literature on resuscitation courses for the above-mentioned populations.

### Limitations

First, we could not meet the original PICOST question as no studies were found that met these criteria. Several of the included studies in this scoping review on BLS education were for individuals with disabilities. However, its aim was not on tailored training but rather on a depiction of CPR training’s feasibility for disabled individuals [20]. In addition, we did not have the opportunity to search grey literature, which might have provided additional insights. Also, we did not assess the whole body of literature on chest-compression-only CPR. Lastly, even though this scoping review covered a topic on specific population groups, we recognize that none of the involved Task Force members or content experts were/are members of the groups we reported on.

## 5. Conclusions

Tailored basic life support education for specific populations is likely feasible and can include such groups into the pool of potential bystander cardiorespiratory resuscitation providers who may otherwise have been left out (e.g., individuals with disabilities). Research should be undertaken to address the identified knowledge gaps, especially comparing tailored vs. standard courses, their cost/benefit ratio, how to best adapt courses, and how to involve members of the respective communities. Also, tailored courses for first responders with and without a duty to respond should be explored, including police officers, firefighters, and lifeguards. 

## Figures and Tables

**Table 1:** Included studies per geographical region in alphabetical order.

Region	No. of Studies	Countries
Asia	1	India (1)—*lower middle*
Europe	7	Austria (1), Italy (1), Slovenia (1), Spain (4)—*all high*
**Total**	**8**	

Included studies per geographical region. Respective income classifications as per the definition of the World Bank [11].

**Table 2:** Included publications describing specifically tailored courses for specific populations (without comparing them to non-tailored courses).

Publication (Author, Year)	Country (Study or Corresponding Author)	Publication Type	Specific Population (Type, n, Age)	Course Adaptations	Assessed Outcomes	Limitations, Comments
Jorge-Soto, 2017 [13]	Spain	Observational non-randomized comparative study (research letter)	Down syndrome; n = 27; 26.4 ± 5.3 years	“Short and simple” course (“short and easy” lecture, “funny” video, hands-on training) tailored to participants with Down syndrome; chest-compression-only CPR	Skill testing after the course; time to defibrillation (74.5 ± 15 s), “defibrillation objective” (reached by 63%), “quality objective” (reached by 47%)	Study compared participants with vs. without Down syndrome, but not a tailored vs. a non-tailored course; focused on AED use; no detailed information available (research letter)
Martinez-Isasi, 2019 [14]	Spain	Observational study (research letter)	Blind; n = 27; age not reported	“Training adapted to the participants’ needs”; chest-compression-only CPR	Skill testing after the course; 74.1% could effectively defibrillate (after 65 ± 27 s). Only 22.2% reached the right compression rate and depth.	No detailed information available (research letter)
Martinez-Isasi, 2021 [15]	Spain	Observational non-randomized comparative study	Blind; n = 29; 53.7 ± 12.3 years	Trainers with special pedagogic training focused on blind people; training under direct supervision by an expert; student/trainer ratio <5/trainer; encouraging tactile contact with the materials; “explanation of the different techniques and steps, considering the blindness of participants”; chest compressions plus rescue breaths	Skill testing after the course; The chain of survival was sufficiently initiated, and chest compressions and rescue breaths were provided. Optimal chest compression depth and compression rate were only achieved by 27.6% and by 48.3% of blind participants, respectively.	Study compared blind vs. blindfolded participants, but not a tailored vs. a non-tailored course
Rodriguez-Nunez, 2015 [16]	Spain	Observational study	Down syndrome; n = 19; 23.3 (no SD reported) years	Adapted course “taking into consideration” a reduced attention span: playful video with comic elements and instructor-led training; chest-compression-only CPR	Skill testing after the course; CPR quality: 20 ± 25% of participants within correct chest compression rate range, 84 ± 31% too shallow, 46 ± 42% with an incomplete release, only 13 ± 18% performed fully correct chest compressions	Study compared participants with vs. without Down syndrome, but not a tailored vs. a non-tailored course
Sandroni, 2004 [12]	Italy	Pre–post study	Deafness; n = 9; no age reported	Initial lecture in sign language (translation provided by an interpreter on site), subsequent training without translation (but using gestures and lip reading); chest compressions plus rescue breaths	Skill testing before and after the course (none of the participants had prior CPR knowledge); safety was checked in 0 vs. 100% (before and after the course, respectively; *p* < 0.001), a shock delivered in 78 vs. 100% (n.s.), the pads placed correctly in 89 vs. 100% (n.s.), and the durations until analysis (80 ± 23.5 vs. 28.9 ± 5.6 s; *p* < 0.001), shock delivery (24.7 ± 4.7 vs. 18.6 ± 1.3 s; *p* = 0.007)-, and the interval between AED on and first shock (101.6 ± 28.4 vs. 47.8 vs. 5.4 s; *p* = 0.001) were shorter after the course	Pre–post comparison, but no comparison of a tailored vs. a non-tailored course; rescue breath assessment not reported
Schnaubelt, 2021 [19]	Austria	Observational study	Refugees; n = 147; 27.5 (22.5–32.5) years	Student/trainer ratio <5/trainer, translators for the native languages on site, initial lecture included basic anatomy and physiology, chest-compression-only CPR	Knowledge testing after the course; willingness to perform CPR increased from 25% before- to 99% after the course (*p* < 0.001). When asked after the course: 98.6% felt better prepared for an emergency, 98.6% would perform CPR in a real situation, 87.1% knew the correct order and process of the chain of survival, 94.6% knew the correct emergency call number, 89.1% knew when to check for breathing, 89.1% knew correct chest compression details; 89.1% knew start and termination rules of BLS; 78.9% knew about the correct use of an AED, 98.0% would teach BLS to others	No skills tested; countries of origin very heterogenous; adults and minors mixed; opinions about before the course only assessed afterwards
Strnad, 2021 [17]	Slovenia	Pre–post study	Deafness; n = 51; 53.6 (no SD reported) years	An occupational medicine specialist modified the BLS and AED protocol to meet the needs of deaf individuals. In brief: Asking another person to call 112 or sending a text message with crucial data / put AED into the visual field and focus on visual prompts; course accompanied by a sign language interpreter; chest compressions plus rescue breaths	Knowledge testing before the course and knowledge plus skill testing afterwards; the sum of correct knowledge answers was higher after the course (3.51 ± 2.22 vs. 42.16 ± 7.22); a correct chest compression rate was achieved by 41.2% of participants, a correct depth by 23%, and only 2% performed 100% correct chest compressions. 49% could provide adequate chest rise ventilations, and 21.6% performed a correct 30:2 approach.	
Unnikrishnan, 2017 [18]	India	Observational study (research letter)	Speech and hearing impairment; n = 6; 23.0 ± 8.1 years	A “special education teacher” proficient in “total communication” on site parallel to the instructors; chest compressions plus rescue breaths	Identification of limitations in applications of the chain of survival for individuals with speech and hearing impairment; activating the EMS and following voice prompts of the AED were perceived as the major points; all participants “accurately” conducted BLS	No knowledge or skills assessment

Data extraction table with the publications grouped in two groups according to tailoring and comparing their course content. AED = automated external defibrillator; BLS = basic life support; CPR = cardiopulmonary resuscitation; SD = standard deviation.

## Data Availability

The data presented in this study are available upon request from the corresponding author.

## Supplementary Materials

The following supporting information can be downloaded at: https://www.mdpi.com/article/10.3390/jcm13144032/s1, Supplement S1: PRISMA statement; Supplement S2: search strategy.

## References

[1] Semeraro F., Greif R., Böttiger B.W., Burkart R., Cimpoesu D., Georgiou M., Yeung J., Lippert F., Lockey A.S., Olasveengen T.M. (2021). European Resuscitation Council Guidelines 2021: Systems saving lives. Resuscitation.

[2] Greif R., Lockey A., Breckwoldt J., Carmona F., Conaghan P., Kuzovlev A., Pflanzl-Knizacek L., Sari F., Shammet S., Scapigliati A. (2021). European Resuscitation Council Guidelines 2021: Education for resuscitation. Resuscitation.

[3] ERC Bringing Resuscitation to the World. https://www.erc.edu/courses/basic-life-support.

[4] Basic Life Support (BLS) CprHeartOrg. https://cpr.heart.org/en/cpr-courses-and-kits/healthcare-professional/basic-life-support-bls-training.

[5] Rott N., Böttiger B.W., Lockey A. (2021). The World Restart a Heart Initiative: How to save hundreds of thousands of lives worldwide. Curr. Opin. Crit. Care.

[6] Schroeder D.C., Semeraro F., Greif R., Bray J., Morley P., Parr M., Nakagawa N.K., Iwami T., Finke S.-R., Hansen C.M. (2023). KIDS SAVE LIVES: Basic Life Support Education for Schoolchildren: A Narrative Review and Scientific Statement from the International Liaison Committee on Resuscitation. Resuscitation.

[7] International Liaison Committee on Resuscitation. https://www.ilcor.org/documents/continuous-evidence-evaluation-guidance-and-templates.

[8] Arksey H., O’Malley L. (2005). Scoping studies: Towards a methodological framework. Int. J. Soc. Res. Methodol..

[9] Tricco A.C., Lillie E., Zarin W., O’Brien K.K., Colquhoun H., Levac D., Moher D., Peters M.D.J., Horsley T., Weeks L. (2018). PRISMA Extension for Scoping Reviews (PRISMA-ScR): Checklist and Explanation. Ann. Intern. Med..

[10] BLS Training in High-Risk Groups. https://costr.ilcor.org/document/bls-training-in-high-risk-groups.

[11] World Bank Country and Lending Groups—World Bank Data Help Desk. https://datahelpdesk.worldbank.org/knowledgebase/articles/906519-world-bank-country-and-lending-groups.

[12] Sandroni C., Fenici P., Franchi M.L., Cavallaro F., Menchinelli C., Antonelli M. (2004). Automated external defibrillation by untrained deaf lay rescuers. Resuscitation.

[13] Jorge-Soto C., Barcala-Furelos R., Gómez-González C., Leborans-Iglesias P., Campos-Varela I., Rodríguez-Núñez A. (2017). Brief training in automated external defibrillation use for persons with down syndrome. Resuscitation.

[14] Martínez-Isasi S., Abelairas-Gómez C., Fernández-Méndez F., Barcala-Furelos R., Jorge-Soto C., Gómez-Gónzalez C., Rodríguez-Nuñez A. (2019). Is it necessary to see to save a life? Pilot study of basic CPR training for blind people. Resuscitation.

[15] Martínez-Isasi S., Jorge-Soto C., Barcala-Furelos R., Abelairas-Gómez C., Carballo-Fazanes A., Fernández-Méndez F., Gómez-González C., Nadkarni V.M., Rodríguez-Núñez A. (2021). Performing Simulated Basic Life Support without Seeing: Blind vs. Blindfolded People. Int. J. Environ. Res. Public Health.

[16] Rodríguez-Núñez A., Regueiro-García A., Jorge-Soto C., Cañas-González J., Leboráns-Iglesias P., García-Crespo O., Barcala-Furelos R. (2015). Quality of chest compressions by Down syndrome people: A pilot trial. Resuscitation.

[17] Strnad M., Šalda Z., Jerko B., Vrečar V., Lesjak V.B., Petrovčič R. (2021). Challenges in basic life support and automated external defibrillator training of deaf individuals. Signa Vitae.

[18] Unnikrishnan R., Babu A.S., Rao P.T., Aithal V., Krishna H.M. (2017). Training individuals with speech and hearing impairment in basic life support: A pilot study. Resuscitation.

[19] Schnaubelt S., Schnaubelt B., Pilz A., Oppenauer J., Yildiz E., Schriefl C., Ettl F., Krammel M., Garg R., Niessner A. (2021). BLS courses for refugees are feasible and induce commitment towards lay rescuer resuscitation. Eur. J. Clin. Investig..

[20] Berlanga-Macías C., Barcala-Furelos R., Méndez-Seijo N., Peixoto-Pino L., Martínez-Isasi S. (2023). Basic life support training for people with disabilities. A scoping review. Resusc. Plus.

[21] Paratz E.D., La Gerche A. (2022). The flatlining of cardiac arrest survival: Can we revive the upward trend?. Eur. Heart J..

[22] Ko Y.-C., Hsieh M.-J., Schnaubelt S., Matsuyama T., Cheng A., Greif R. (2023). Disparities in layperson resuscitation education: A scoping review. Am. J. Emerg. Med..

[23] Berg K.M., Cheng A., Panchal A.R., Topjian A.A., Aziz K., Bhanji F., Bigham B.L., Hirsch K.G., Hoover A.V., Kurz M.C. (2020). Part 7: Systems of Care: 2020 American Heart Association Guidelines for Cardiopulmonary Resuscitation and Emergency Cardiovascular Care. Circulation.

[24] Schnaubelt S., Garg R., Atiq H., Baig N., Bernardino M., Bigham B., Dickson S., Geduld H., Al-Hilali Z., Karki S. (2023). Cardiopulmonary resuscitation in low-resource settings: A statement by the International Liaison Committee on Resuscitation, supported by the AFEM, EUSEM, IFEM, and IFRC. Lancet Glob. Health.

[25] Elgohary M., Palazzo F., Breckwoldt J., Cheng A., Pellegrino J., Schnaubelt S., Greif R., Lockey A. (2022). Blended learning for accredited life support courses—A systematic review. Resusc. Plus.

[26] Schnaubelt S., Monsieurs K., Semeraro F., Schlieber J., Cheng A., Bigham B., Garg R., Finn J., Greif R., Bray J. (2020). Clinical outcomes from out-of-hospital cardiac arrest in low-resource settings—A scoping review. Resuscitation.

[27] Disparity in Layperson Resuscitation Education: A Task Force Scoping Review (EIT 6102). https://costr.ilcor.org/document/disparity-in-layperson-resuscitation-education-a-task-force-scoping-review-eit-6102.

[28] Dobbie F., Uny I., Eadie D., Duncan E., Stead M., Bauld L., Angus K., Hassled L., MacInnes L., Clegg G. (2020). Barriers to bystander CPR in deprived communities: Findings from a qualitative study. PLoS ONE.

[29] Abdulhay N.M., Totolos K., McGovern S., Hewitt N., Bhardwaj A., Buckler D.G., Leary M., Abella B.S. (2019). Socioeconomic disparities in layperson CPR training within a large U.S. city. Resuscitation.

[30] Birkun A., Kosova Y. (2018). Social attitude and willingness to attend cardiopulmonary resuscitation training and perform resuscitation in the Crimea. World J. Emerg. Med..

[31] Fratta K.A., Bouland A.J., Lawner B.J., Comer A.C., Halliday M.H., Levy M.J., Seaman K.G. (2019). Barriers to bystander CPR: Evaluating socio-economic and cultural factors influencing students attending community CPR training. Am. J. Emerg. Med..

[32] Fratta K.A., Bouland A.J., Vesselinov R., Levy M.J., Seaman K.G., Lawner B.J., Hirshon J.M. (2020). Evaluating barriers to community CPR education. Am. J. Emerg. Med..

[33] Naim M.Y., Griffis H.M., Burke R.V., McNally B.F., Song L., Berg R.A., Nadkarni V.M., Vellano K., Markenson D., Bradley R.N. (2019). Race/Ethnicity and Neighborhood Characteristics Are Associated with Bystander Cardiopulmonary Resuscitation in Pediatric Out-of-Hospital Cardiac Arrest in the United States: A Study from CARES. J. Am. Heart Assoc..

[34] Uber A., Sadler R.C., Chassee T., Reynolds J.C. (2017). Bystander Cardiopulmonary Resuscitation Is Clustered and Associated with Neighborhood Socioeconomic Characteristics: A Geospatial Analysis of Kent County, Michigan. Acad. Emerg. Med..

[35] Birkun A., Trunkwala F., Gautam A., Okoroanyanwu M., Oyewumi A. (2020). Availability of basic life support courses for the general populations in India, Nigeria and the United Kingdom: An internet-based analysis. World J. Emerg. Med..

[36] Husain S., Eisenberg M. (2013). Police AED programs: A systematic review and meta-analysis. Resuscitation.

[37] Stein P., Spahn G.H., Müller S., Zollinger A., Baulig W., Brüesch M., Seifert B., Spahn D.R. (2017). Impact of city police layperson education and equipment with automatic external defibrillators on patient outcome after out of hospital cardiac arrest. Resuscitation.

[38] White R.D., Asplin B.R., Bugliosi T.F., Hankins D.G. (1996). High discharge survival rate after out-of-hospital ventricular fibrillation with rapid defibrillation by police and paramedics. Ann. Emerg. Med..

[39] Høyer C.B., Christensen E.F. (2009). Fire fighters as basic life support responders: A study of successful implementation. Scand. J. Trauma Resusc. Emerg. Med..

[40] Louis C.J., Cildoz M., Echarri A., Beaumont C., Mallor F., Greif R., Baigorri M., Reyero D. (2023). Police as first reponders improve out-of-hospital cardiac arrest survival. BMC Emerg. Med..

[41] Cho B.-J., Kim S.-R. (2021). Comparison of Long-Term Effects between Chest Compression-Only CPR Training and Conventional CPR Training on CPR Skills among Police Officers. Healthcare.

[42] Ross P., Nolan J., Hill E., Dawson J., Whimster F., Skinner D. (2001). The use of AEDs by police officers in the City of London. Automated external defibrillators. Resuscitation.

[43] Abelairas-Gómez C., Rodríguez-Núñez A., Greif R. (2021). Now it is time to teach to schoolteachers: The long road to the Schoolteacher BLS Teaching Curriculum. Resuscitation.

[44] Abelairas-Gómez C., Schroeder D.C., Carballo-Fazanes A., Böttiger B.W., López-García S., Martínez-Isasi S., Rodríguez-Núñez A. (2021). KIDS SAVE LIVES in schools: Cross-sectional survey of schoolteachers. Eur. J. Pediatr..

[45] Iserbyt P., Schouppe G., Charlier N. (2015). A multiple linear regression analysis of factors affecting the simulated Basic Life Support (BLS) performance with Automated External Defibrillator (AED) in Flemish lifeguards. Resuscitation.

[46] Claesson A., Karlsson T., Thorén A.-B., Herlitz J. (2011). Delay and performance of cardiopulmonary resuscitation in surf lifeguards after simulated cardiac arrest due to drowning. Am. J. Emerg. Med..

[47] Seesink J., Nieuwenburg S.A.V., van der Linden T., Bierens J.J.L.M. (2019). Circumstances, outcome and quality of cardiopulmonary resuscitation by lifeboat crews. Resuscitation.

[48] Barcala-Furelos R., Aranda-García S., Otero-Agra M., Fernández-Méndez F., Alonso-Calvete A., Martínez-Isasi S., Greif R., Rodríguez-Núñez A. (2023). Are smart glasses feasible for dispatch prehospital assistance during on-boat cardiac arrest? A pilot simulation study with fishermen. Intern. Emerg. Med..

[49] Hawkes C.A., Brown T.P., Booth S., Fothergill R.T., Siriwardena N., Zakaria S., Askew S., Williams J., Rees N., Ji C. (2019). Attitudes to Cardiopulmonary Resuscitation and Defibrillator Use: A Survey of UK Adults in 2017. J. Am. Heart Assoc..

[50] Krammel M., Schnaubelt S., Weidenauer D., Winnisch M., Steininger M., Eichelter J., Hamp T., van Tulder R., Sulzgruber P. (2018). Gender and age-specific aspects of awareness and knowledge in basic life support. PLoS ONE.

[51] Dolenc E., Slabe D., Kovačič U. (2021). The needs and opportunities of older laypeople to acquire first aid skills. PLoS ONE.

[52] Hassounah M.M., AlOwaini H.S., Diab C.N., Khamis N.N. (2018). YouTube videos teaching Arabic speaking population how to perform cardiopulmonary resuscitation: The gap between the need and quality!. Resuscitation.

[53] Berg I., Haveman B., Markovic O., van de Schoot D., Dikken J., Goettinger M., Peden A.E. (2021). Characteristics of surfers as bystander rescuers in Europe. Am. J. Emerg. Med..

[54] (2023). UEFA.com. Brazil and England’s Teams CPR Trained Ahead of Women’s Finalissima|Inside UEFA. https://www.uefa.com/insideuefa/news/0280-17b46b07f5ef-390ef5aef76d-1000--brazil-and-england-s-teams-cpr-trained-ahead-of-women-s-f/.

[55] Lott C., van Goor S., Nikolaou N., Thilakasiri K., Bahtijarević Z. (2023). “Get trained. Save lives.”: A CPR awareness campaign in football. Resuscitation.

[56] Kim J.H., Malhotra R., Chiampas G., D’Hemecourt P., Troyanos C., Cianca J., Smith R.N., Wang T.J., Roberts W.O., Thompson P.D. (2012). Cardiac arrest during long-distance running races. N. Engl. J. Med..

[57] Hinkelbein J., Kerkhoff S., Adler C., Ahlbäck A., Braunecker S., Burgard D., Cirillo F., De Robertis E., Glaser E., Haidl T.K. (2020). Cardiopulmonary resuscitation (CPR) during spaceflight—A guideline for CPR in microgravity from the German Society of Aerospace Medicine (DGLRM) and the European Society of Aerospace Medicine Space Medicine Group (ESAM-SMG). Scand. J. Trauma Resusc. Emerg. Med..

[58] Hinkelbein J., Böhm L., Braunecker S., Genzwürker H.V., Kalina S., Cirillo F., Komorowski M., Hohn A., Siedenburg J., Bernhard M. (2018). In-flight cardiac arrest and in-flight cardiopulmonary resuscitation during commercial air travel: Consensus statement and supplementary treatment guideline from the German Society of Aerospace Medicine (DGLRM). Intern. Emerg. Med..

[59] Chilappa R., Waxman M.J. (2021). Basic Life Support Awareness and Knowledge in High School Students. Kans. J. Med..

[60] Baldi E., Savastano S., Contri E., Lockey A., Conaghan P., Hulme J., Cimpoesu D., Maconochie I., Böttiger B.W., Greif R. (2020). Mandatory cardiopulmonary resuscitation competencies for undergraduate healthcare students in Europe: A European Resuscitation Council guidance note. Eur. J. Anaesthesiol..

[61] Hooker E.A., O’Brien D. (2007). Ambulance transports from a state prison: A unique service staffed by prisoners. J. Ky. Med. Assoc..

