# Supplementary material for: Tailored Basic Life Support Training for Specific Layperson Populations—A Scoping Review
Source: J Clin Med. 2024 Jul 10;13(14):4032. doi: 10.3390/jcm13144032 (PMC11277549; doi:10.3390/jcm13144032)
Supplement: Supplementary file 1 [file jcm-13-04032-s001.zip › Supplement S2.pdf]

Supplement S2: Search Strategy

Database Name	Number of items Identified	Number of items (Duplicates Removed)
Medline	978	973
Embase	1022	176
CCRT	178	54
Total – All Sources	2178	1203

Search Strategies

Embase 1974 to 2023 July 07, Ovid MEDLINE(R) ALL 1946 to July 06, 2023

1	Resuscitation/ or Cardiopulmonary Resuscitation/ or Heart Massage/ or Heart Arrest/ or "Out-of-Hospital Cardiac Arrest"/ or cardiopulmonary arrest/	270813
2	(resuscitat* or ((cardiac or heart) adj2 (massag* or compression*)) or (chest adj2 compression*) or CPR or "basic life support" or "basic cardiac life support" or BCLS or BLS or "automated external defibrillator*" or "automatic external defibrillator*" or AED or AEDs or "cardiac arrest").ti,ab,kf,kw.	310176
3	1 or 2 [RESUSCITATION]	412903
4	(bystander* or by-stander* or layperson* or layman or laymen or laywoman or laywomen or "lay person*" or "lay man" or "lay men" or "lay people" or "public setting*").ti,ab,kf,kw.	45093
5	(("non healthcare" or "non health care" or "non medical") adj3 "first responder*").ti,ab,kf,kw.	23
6	Police/ or "Law Enforcement"/ or Firefighters/ or "School Teachers"/ or fire fighter/ or school teacher/ or exp airplane crew/	53256
7	("law enforcement" or police or firefighter* or fire-fighter* or "life guard*" or lifeguard* or "flight crew*" or "flight attendant*" or teacher* or "visitation service*" or "visitation program*" or "duty to attend").ti,ab,kf,kw.	184497
8	or/4-7 [LAYPERSONS]	250541
9	3 and 8 [RESUSCITATION + LAYPERSONS]	10779
10	(Education/ or "Education, Nonprofessional"/ or Inservice Training/ or Teaching/ or "in service training"/) and (tailor* or conceptualiz* or conceptualis* or adapted or adaptation or adjusted or adjustment or customized or customised or "custom made" or implementation).ti,ab,kf,kw.	80274
11	((tailor* or conceptualiz* or conceptualis* or adapted or adaptation or adjusted or adjustment or customized or customised or "custom made" or novel or specific or developed or development or implementation) and (educat* or train* or course* or ((knowledge or skill or skills) adj3 (acquisition or aquir*)))).ti,ab,kf,kw.	1744823
12	10 or 11 [TAILORED TRAINING]	1755953
13	9 and 12 [RESUSCITATION + LAYPERSONS + TAILORED TRAINING]	1430

14	"Health Knowledge, Attitudes, Practice"/ or Socioeconomic Factors/ or Social Class/ or "attitude to health"/ or socioeconomic/	685450
15	(barrier* or "deprived communit*" or socioeconomic or "socio economic" or SES or "low resource*" or resources or cultural or willingness or satisfaction or retention or feasibility).ti,ab,kf,kw.	3605354
16	14 or 15 [ADDITIONAL FACTORS]	4065967
17	9 and 16 [RESUSCITATION + LAYPERSONS + ADDITIONAL FACTORS]	1796
18	13 or 17 [(RESUSCITATION + LAYPERSONS + TAILORED TRAINING) OR (RESUSCITATION + LAYPERSONS + ADDITIONAL FACTORS)]	2771
19	(Animals/ or "Animal Experimentation"/ or "Models, Animal"/ or "Disease Models, Animal"/) not (Humans/ or "Human Experimentation"/)	9002167
20	(exp "animal model"/ or exp "animal experiment"/ or "nonhuman"/ or exp "vertebrate"/) not (exp "human"/ or exp "human experiment"/)	11247874
21	18 not (19 or 20) [ANIMAL STUDIES REMOVED]	2756
22	(comment or editorial or "newspaper article" or news or note or lecture).pt.	3420501
23	(letter not (letter and randomized controlled trial)).pt.	2527266
24	21 not (22 or 23) [OPINION PIECES REMOVED]	2728
25	(conference or conference abstract or "conference review" or congresses).pt.	5586693
26	24 not 25 [CONFERENCES REMOVED]	2034
27	Case Reports.pt. or case report/ or exp case study/	5343582
28	26 not 27 [CASE REPORTS REMOVED]	2005
29	limit 28 to english language	1865
30	limit 28 to abstracts	1992
31	29 or 30 [ENGLISH LANGUAGE OR ENGLISH ABSTRACTS]<br>Embase <1974 to 2023 July 07><br>Ovid MEDLINE(R) ALL <1946 to July 06, 2023>	2000<br>1022<br>978
32	remove duplicates from 31<br>Embase <1974 to 2023 July 07><br>Ovid MEDLINE(R) ALL <1946 to July 06, 2023>	1166<br>193<br>973

Cochrane Central Register of Controlled Trials via Cochrane Library Wiley Online Issue 7 of 12, July 2023

#1	(resuscitat* or ((cardiac or heart) NEAR/2 (massag* or compression*)) or (chest NEAR/2 compression*) or CPR or "basic life support" or "basic cardiac life support" or BCLS or BLS or "automated external defibrillator" or "automatic external defibrillator" or "automated external defibrillators" or "automatic external defibrillators" or AED or AEDs or "cardiac arrest"):ti,ab,kw	14200
#2	(bystander* or by-stander* or layperson* or layman or laymen or laywoman or laywomen or "lay person" or "lay persons" or "lay man" or "lay men" or "lay people" or "public setting" or "public settings"):ti,ab,kw	1259

#3	(("non healthcare" or "non health care" or "non medical") NEAR/3 ("first responder" or "first responders")):ti,ab,kw	2
#4	("law enforcement" or police or firefighter* or fire-fighter* or "life guard" or "life guards" or lifeguard* or "flight crew" or "flight attendant" or "flight crews" or "flight attendants" or teacher* or "visitation service" or "visitation program" or "visitation services" or "visitation programs" or "visitation programme" or "visitation programmes" or "duty to attend"):ti,ab,kw	8473
#5	{OR #2-#4}	9675
#6	((tailor* or conceptualiz* or conceptualis* or adapted or adaptation or adjusted or adjustment or customized or customised or "custom made" or novel or specific or developed or development or implementation) and (educat* or train* or course* or ((knowledge or skill or skills) adj3 (acquisition or aquir*)))):ti,ab,kw	96182
#7	#1 and #5 and #6	124
#8	(barrier* or "deprived community" or "deprived communities" or socioeconomic or "socio economic" or SES or "low resource" or "low resources" or resources or cultural or willingness or satisfaction or retention or feasibility):ti,ab,kw	184379
#9	#1 and #5 and #8	130
#10	#7 or #9	216
#11	([mh ^Animals] OR [mh ^"Animal Experimentation"] OR [mh ^"Models, Animal"] OR [mh ^"Disease Models, Animal"]) NOT ([mh ^Humans] OR [mh ^"Human Experimentation"])	2674
#12	#10 not #11	216
#13	conference proceeding:pt	224781
#14	#12 not #13	180
#15	#12 not #13 in Trials	178
